# Mena deficiency delays tumor progression and decreases metastasis in polyoma middle-T transgenic mouse mammary tumors

**DOI:** 10.1186/bcr2784

**Published:** 2010-11-25

**Authors:** Evanthia T Roussos, Yarong Wang, Jeffrey B Wyckoff, Rani S Sellers, Weigang Wang, Jiufeng Li, Jeffrey W Pollard, Frank B Gertler, John S Condeelis

**Affiliations:** 1 Department of Anatomy and Structural Biology, Albert Einstein College of Medicine, 1301 Morris Park Avenue, Bronx, NY 10461, USA; 2 Gruss Lipper Biophotonics Center, Albert Einstein College of Medicine, 1301 Morris Park Avenue, Bronx, NY 10461, USA; 3 Department of Pathology, Albert Einstein College of Medicine, 1301 Morris Park Avenue, Bronx, NY 10461, USA; 4 Department of Developmental and Molecular Biology, Albert Einstein College of Medicine, 1300 Morris Park Avenue, Bronx, NY 10461, USA; 5 David H Koch Institute for Integrative Cancer Research, Massachusetts Institute of Technology, Koch Institute, 77 Massachusetts Avenue, Cambridge, MA 02139, USA

## Abstract

**Introduction:**

The actin binding protein Mammalian enabled (Mena), has been implicated in the metastatic progression of solid tumors in humans. Mena expression level in primary tumors is correlated with metastasis in breast, cervical, colorectal and pancreatic cancers. Cells expressing high Mena levels are part of the tumor microenvironment for metastasis (TMEM), an anatomical structure that is predictive for risk of breast cancer metastasis. Previously we have shown that forced expression of Mena adenocarcinoma cells enhances invasion and metastasis in xenograft mice. Whether Mena is required for tumor progression is still unknown. Here we report the effects of Mena deficiency on tumor progression, metastasis and on normal mammary gland development.

**Methods:**

To investigate the role of Mena in tumor progression and metastasis, Mena deficient mice were intercrossed with mice carrying a transgene expressing the polyoma middle T oncoprotein, driven by the mouse mammary tumor virus. The progeny were investigated for the effects of Mena deficiency on tumor progression via staging of primary mammary tumors and by evaluation of morbidity. Stages of metastatic progression were investigated using an *in vivo *invasion assay, intravital multiphoton microscopy, circulating tumor cell burden, and lung metastases. Mammary gland development was studied in whole mount mammary glands of wild type and Mena deficient mice.

**Results:**

Mena deficiency decreased morbidity and metastatic dissemination. Loss of Mena increased mammary tumor latency but had no affect on mammary tumor burden or histologic progression to carcinoma. Elimination of Mena also significantly decreased epidermal growth factor (EGF) induced *in vivo *invasion, *in vivo *motility, intravasation and metastasis. Non-tumor bearing mice deficient for Mena also showed defects in mammary gland terminal end bud formation and branching.

**Conclusions:**

Deficiency of Mena decreases metastasis by slowing tumor progression and reducing tumor cell invasion and intravasation. Mena deficiency during development causes defects in invasive processes involved in mammary gland development. These findings suggest that functional intervention targeting Mena in breast cancer patients may provide a valuable treatment option to delay tumor progression and decrease invasion and metastatic spread leading to an improved prognostic outcome.

## Introduction

Metastasis is the primary cause of death from breast cancer, the most common form of cancer affecting women in the United States, and the second leading cause of cancer related deaths in women around the world [1]. Tumor cells make use of different cellular processes to execute the steps of metastasis: invasion, intravasation, extravasation, tumor cell dissemination, extravasation and growth of distant metastases [1]. Use of multiphoton intravital microscopy enabled the observation of migration of tumor cells across the endothelial barrier into blood vessels at sites containing at least one peri-vascular macrophage [2]. This led to the development of an *in vivo *invasion assay used to capture live, invasive and metastatic tumor cells from the primary tumor for analysis [3]. Furthermore, the study of invasion, intravasation and metastasis using both methods has shown the involvement of a paracrine loop between macrophages and tumor cells that secrete EGF or colony stimulating factor-1 (CSF1), respectively [3,4].

Expression profiling of invasive tumor cells captured using the *in vivo *invasion assay revealed the upregulation of several key cytoskeletal regulatory proteins involved in cell motility [5-10]. Mena was among the proteins that comprise what was termed the Invasion Signature and has since been shown to be upregulated in rat, mouse and human mammary tumors and to correlate with metastatic risk [5,10-12]. Evaluation of high risk primary and metastatic cervical, colorectal and pancreatic cancers have also shown enhanced expression of Mena as compared to low risk cases [11,13-15]. Mena has been used in the development of a prognostic marker of hematogenous metastasis called TMEM (Tumor Micro-Environment for Metastasis), shown to be associated with risk of metastasis in breast cancer patients independently of traditional prognostic markers [12].

Mena (also known as ENAH-enabled homolog) is a member of the Ena/VASP family of actin-regulatory proteins that function in multiple different cell types to regulate cell morphology and motility [16,17]. Similar to other Ena/VASP proteins, Mena regulates the geometry and assembly of actin filament networks through binding of profilin and both G- and F-actin, the ability to promote filament elongation through monomer delivery and anti-capping activity [18,19], and the ability to reduce the density of Arp2/3-mediated branching [17,19-22].

There are at least two alternative splice variants of Mena in tumor cells; the two best characterized isoforms are Mena invasive (^INV^), found exclusively in invasive tumor cells and Mena11a, found in primary tumor cells but lost in invasive cells [5]. Recent studies in rodent models show an increase in metastasis upon forced expression of Mena and Mena^INV ^without any affect on primary tumor growth [23]. This increase in metastasis was found to arise from increased EGF-induced invasion, tumor cell protrusion and matrix degradation activity by invadopodia [23].

To determine if Mena is required for tumor progression and metastasis, we used previously generated Mena Null mice [24] and intercrossed them with transgenic mice carrying the mammary tumor virus (MMT-V)-polyoma middle T antigen (PyMT) [25]. Mammary tumors growing in the PyMT expressing mice go through distinct morphologic stages of tumor progression comparable to progression of human breast disease and develop spontaneous metastases following disease progression [26]. Thus, they provide an excellent model for the investigation of the role of Mena in tumor progression and metastasis.

All Mena isoforms are completely eliminated in Mena homozygous mice and reduced in heterozygote mice [24]. Mena homozygous mutant (Mena Null) mice are fully viable, but slightly smaller than their littermates until adulthood [24]. Mena Null mice exhibit a variety of subtle nervous system defects such as misrouted axon fiber tracts in the cortex including the major brain commissures, the corpus callosum and hippocampus; these phenotypes all become more severe and other structures such as the optic nerve and spinal nerves become disrupted when the other two Ena/VASP paralogs are deleted along with Mena [24,27-29]. The invertebrate orthologs of Mena have been shown to function downstream of axonal guidance receptors DCC and Robo [30,31], which have also been implicated in the morphogenesis of the mammary gland [32]. Little, however, is known about the role of Mena in the developing mammary gland. Since tumor cells are thought to acquire the ability to invade surrounding stroma via a re-awakening of developmental processes [33-35], the investigation of Mena in mammary gland development could provide insight into Mena's role in tumor cell invasion and dissemination in mammary tumors.

We provide evidence that, in Mena Null mice, overall tumor burden is unaffected while tumor latency is increased and mortality is decreased. Additionally, we show that in Mena Null mice, invasion, intravasation, motility and metastasis are significantly decreased. Deficiency of Mena also leads to decreased terminal end bud formation and branching during mammary gland development but has no affect on ductal growth. These findings provide important insight into the functions of Mena during tumor progression and metastasis and provide evidence that these malignant processes could represent the revival of Mena's role in processes resembling mammary gland development within the primary tumor. Our study provides evidence that inhibition of Mena activity may be a useful approach to prevention of metastasis.

## Materials and methods

### Animal models and assays for tumor progression

All studies were carried out using protocols approved by the Albert Einstein College of Medicine Animal Care and Use Committee. GFP labeled macrophages were recreated on the FVB Tg(*Csf1r-eGFP)1 *background using an identical construct and strategy as published [36]. CFP labeled mammary carcinoma cells were generated on the FVB background by co-injection of Tg(*MMTV-iCRE)1jwp *together with *Tg(PCAG-loxp-CAT-stop-loxp*-*CFP*) plasmids by conventional methods in the Albert Einstein Transgenic Mouse Facility and were similar to previously described mice [37]. These mice were then crossed with previously described Mena heterozygotes [24]. Germline transmission of alleles was verified by PCR using the following primer sequences: *MMTV-iCRE-PCAG-loxp-CAT-stop-loxp-CFP*: (forward) CAG GGC CTT CTC CAC ACC AGC, (reverse) CTG GCT GTG AAG ACC ATC, cFMS-GFP: (forward) TCA TTC CAG AAC CAG AGC, (reverse) TGC TCA GGT AGT GGT TGT CG. Primers sequences used to identify transmission of the disrupted Mena allele can be provided upon request. All PyMT mouse tumors were palpated beginning at six weeks of age and measured by calipers to monitor tumor formation.

Orthotopic PyMT xenograft tumors were derived from subcutaneous injection of 1 × 10^6 ^CFP-PyMT primary tumor cells FAC sorted from PyMT/Mena wild type (WT), PyMT/Mena heterozygote (Het) and PyMT/Mena homozygous (Null) mice into the mammary gland of five-to-seven-week-old female severe combined immune deficient (SCID) mice purchased from the National Cancer Institute [3]. The purpose of this study was to confirm that the PyMT tumor cells injected into SCID mice (all with an identical genotype) that were also wild type for *Mena*, would grow and metastasize in a manner similar to that identified in their parental mouse strain. Once tumors reached 2 cm^3 ^in size (approximately 8 to 12 weeks after injection) animals were used for the indicated experiments.

Orthotopic MTLn3 xenograft tumors were derived from mammary gland injection of 1 × 10^6 ^MTLn3 rat adenocarcinoma cells or MTLn3 cells forced to express Mena, into SCID mice [23,38]. Once tumors reached 2 cm^3 ^in size (approximately four weeks after injection) animals were used for the indicated experiments. MTLn3-Mena expressing cells expressed the Mena protein four-fold over endogenous levels to mimic Mena over-expression previously identified in invasive tumor cells [8]. For a full description of cell line derivation, clonal selection and culturing of these cell lines see reference [23].

Staging of mammary tumors from PyMT mice was done according to criteria previously described [26]. Tumors growing in PyMT mice have been shown to progress at different rates, for example at 10 weeks of age it is common to find some tumors that are hyperplastic while other tumors from the same animal have already progressed to invasive carcinoma [26]. Additionally, even within the same tumor there can be regions of that tumor that are normal and regions that show varied histological progression [26]. Therefore, we staged multiple different tumors and different regions within each tumor from each animal evaluated (15 to 20 of each PyMT Mena WT, Het and Null mice) at 10 weeks of age to ensure identification of the most progressed histology. Tumor stage was finally determined and classified based on the most advanced histological stage identified, (that is, a mouse with regions of hyperplasia and invasive carcinoma either within the same tumor or in different tumors, the stage of progression was reported as carcinoma). Evaluation of staging was performed by double blind. To determine the number of tumor cells in circulation, blood was drawn from the right ventricle of the heart in anesthetized mice harboring at least one tumor that was 2 cm^3 ^in size as previously described [39]. Single tumor cells were counted seven days after plating; all cells counted were CFP positive.

### Metastasis characterization and Immunohistochemistry (IHC)

Mammary tumors, mammary glands and lungs were fixed in 10% buffered formalin and routinely embedded in paraffin. Spontaneous lung metastases (>2 mm) were evaluated in formalin fixed, paraffin embedded and hematoxylin and eosin stained lung tissue. To quantify lung metastases, eight 10-μm paraffin sections were cut at 50 μm apart and placed on slides for further staining. Lung metastases were counted in all five lung lobes. Metastases spanning multiple sections were only counted in the first section where they were identified. Evaluation of metastases was performed double blind. Fixed mammary tumors matched for age and tumor stage were stained for vasculature using an antibody against CD-34 (1:100) (Invitrogen, Carlsbad, CA, USA), five 200× fields were analyzed in five animals per genotype to determine differences in vascular density. Fixed mammary glands stained with F4/80 at 1:50 (a gift from Dr. Richard Stanley) to identify macrophages as previously described [40]. F4/80 has been shown to specifically identify mononuclear mouse macrophages [41] and has been used in IHC and FAC sorting to identify macrophages by many different groups [42-44]. Evaluation of the expression of Mena in mammary glands was evaluated in formalin fixed, paraffin embedded tissue from six-week-old PyMT negative Mena WT, Het and Null mice. Primary antibody against Mena was used at (1:1000) [45], antigen retrieval was done using buffered citrate, and DAB (Invitrogen) was used to visualize staining, hematoxylin was used as a counterstain. All slides were imaged using Zeiss AxioObserver.Z1 5× DIC1, EC Plan-Neofluar 10×/0.3 Ph1, EC Plan-Neofluar 20×/0.5 Ph2 M27, EC Plan-Neofluar 40×/0.75 Ph2 M27, EC Plan-neofluar 63×/1.4 Oil and an AxioCamHR3. All objectives and microscopes were manufactured by Zeiss, Oberkochen, Germany.

### Whole mount mammary gland preparation and staining

The evaluation of mammary gland maturation was performed on five of each PyMT negative Mena WT, Het, and Null mice at 6 and 10 weeks of age. Following euthanasia, the fourth abdominal mammary glands were surgically removed and placed on a glass slide and fixed and stained as previously described [40]. Ductal length, terminal end bud counts and branching were determined as previously described [40]. To determine normalized macrophages/TEB area, macrophages were counted around individual TEBs and the area of each of these TEBs was measured. Data represent the number of macrophages/TEB area in pixels and are normalized to a constant area. To determine the average number of macrophages/area, macrophages were counted within an identical fixed area either surrounding TEBs or in stroma (without any TEBs). Data represent raw counts of macrophages within this fixed area. A minimum of 10 fields were counted from five mice per genotype for these experiments.

### Intravital imaging

Intravital multiphoton imaging was performed on 15 of each PyMT Mena WT, Het and Null mice as described previously using a 20× 1.95 NA water immersion objective with correction lens [46,47]. Time-lapse movies were analyzed for frequency of motility in three dimensions and through time using ImageJ [48]. A cell movement event was defined as a translocation ≥1 cell diameter within a field, a field is defined as 100 μm *512 × 512* minute. To acquire this size volume took two minutes. All movies of cell movements were 30 minutes in duration.

### *In vivo* invasion assay

The *in vivo *invasion assay was performed in 5 to 10 mice per condition as previously described [49]. Briefly, needles were held in place by a micromanipulator around a single mammary tumor of an anesthetized mouse. Needles contained a mixture of Matrigel™, 25 nM EGF, buffer and EDTA. After four hours of cell collection the contents of the needles were extruded. Cells were then stained with DAPI and counted using an Olympus IX70 inverted microscope with a 10× NA 0.30 objective.

### Statistical analysis

Statistical significances were determined using unpaired, two-tailed Student's *t*-tests assuming equal variances and an alpha level of 0.05 for all experiments unless otherwise indicated. For lung metastasis and assays evaluating the number of circulating tumor cells in the blood non-parametric the Mann Whitney Wilcoxon rank sum test was used. For Kaplan-Meier plots Log-rank (Mantel-Cox) t-test was performed.

## Results

### Deficiency of Mena increases tumor latency and decreases morbidity

**Figure 1:**
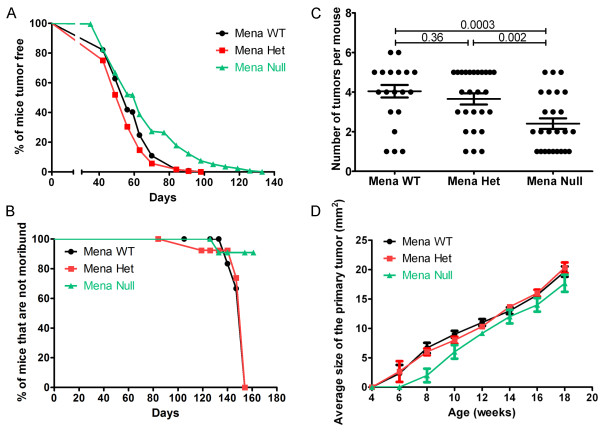
**Deficiency of Mena increases tumor latency and decreases morbidity**. **A**. Percent of mice without palpable tumors at the given age. N >100 mice/genotype. *P*-values by Log-rank (Mantel-Cox) test: Mena WT vs. Het = 0.02, Mena WT vs. Null <0.0001, Mena Het vs. Null <0.0001. **B**. Kaplan Meir curve measuring percentage of mice from each genotype that are not moribund (mice that have not yet developed tumors or mice that have small tumors that have not immobilized them). N = 12 to 25 mice/genotype. *P*-values by Log-rank (Mantel-Cox) test: Mena WT vs. Het = 0.44, Mena WT vs. Null = 0.01, Mena Het vs. Null = 0.05. **C**. Average number of tumors per mouse at 10 weeks of age. N = 25 mice/genotype. Error bars indicate SEM. *P*-values by student's *t*-test are listed above columns. **D**. Average tumor size at given age. Tumors measured with digital calipers. For each genotype N = 5 mice/given age. Error bars indicate SEM. *P*-values by student's *t*-test for Mena WT vs. Mena Null at 8 weeks = 0.033, at 10 weeks = 0.034, all other *P*-values > 0.05.

We sought to determine whether MMTV-PyMT-driven mammary tumor formation is affected by the absence of Mena. Investigation of mammary tumor onset showed that Mena deficiency increased tumor latency significantly (Figure 1A) compared to Mena WT and Het mice (*P*-value = <0.0001). Mice heterozygous for Mena showed a slight decrease in tumor latency as compared to WT mice (Figure 1A) (*P*-value = 0.02).

Mice were considered 'not moribund' until they either died or had to be euthanized due to illness or immobilization as a result of their tumor burden. Mena Null mice had a later tumor onset than did the Mena WT or Het mice (Figure 1A) and survived longer than either Mena WT or Het mice that reached the tumor size limit or died (*P-*value = 0.01, 0.03 respectively) (Figure 1B). There were no significant differences in morbidity between Mena WT and Het mice (*P-*value = 0.91) (Figure 1B).

In those mice with tumors at 10 weeks of age, Mena Null mice had significantly fewer tumors/animal (Figure 1C). Additionally, tumor growth was significantly decreased in Mena Null mice that were growing tumors at both 8 and 10 weeks of age. These results reflect the increased tumor latency in Mena Null mice observed between 60 and 100 days as shown in Figure 1A. Interestingly, as Mena Null mice aged, the number of tumors per animal as well as tumor growth were not significantly different as compared to Mena WT or Het mice (Figure S1 in Additional file 1 Figure 1D).

### Deficiency of Mena slows progression to invasive carcinoma

**Figure 2:**
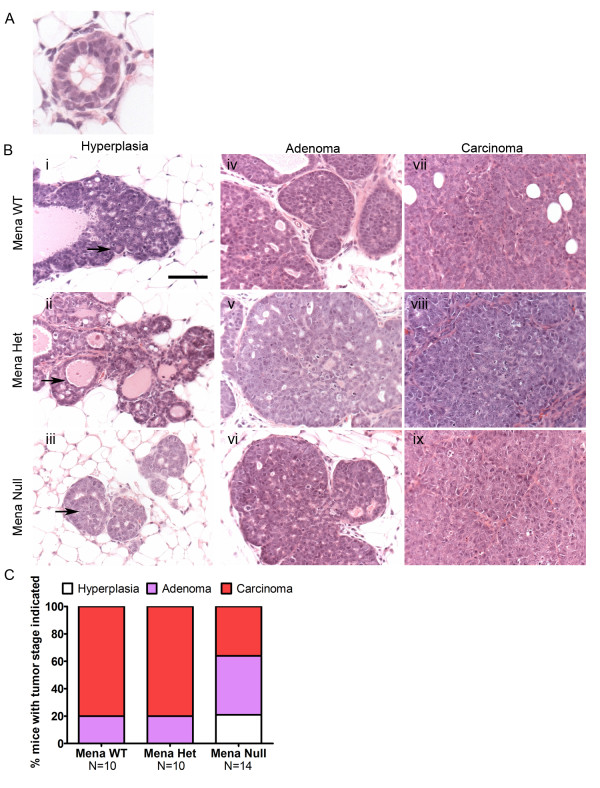
**Deficiency of Mena slows progression to invasive carcinoma**. **A**. Immunohistochemistry of normal mammary acinus from PyMT mouse at 10 weeks of age stained with Hematoxylin and Eosin. **B**. Immunohistochemistry of mammary gland tumors from PyMT Mena WT, Het and Null mice at 10 weeks of age stained with Hematoxylin and Eosin. Mammary glandular hyperplasia (i, ii, iii); Acini are not notably expanded in size (arrow). Mammary gland adenomas (iv, v, vi). Mammary carcinomas (vii, viii, ix). Scale bar = 100 μm. High magnification images showing some of the characteristics used to stage tumors are shown in Figure S2 in Additional file 1. **C**. Percent of mice with hyperplastic changes (white bar), adenoma (purple bar), and carcinoma (red bar). N = number of mice used for each genotype (listed below the x-axis).

Distinct stages of tumor progression have been identified in PyMT-generated mammary tumors and have been shown to correlate with the benign, *in situ *proliferative lesions, and invasive carcinomas seen in humans [26]. We used a classification system that identifies distinct histopathologic changes and represents morphological events of tumor progression from benign to malignant: hyperplasia, adenoma and invasive carcinoma [26]. Characteristics used to determine stage of tumor progression include appearance of acini, individual epithelial cell morphology and structure of the mammary glands (Figure 2A, B and Figure S2 in Additional file 1). Mammary glandular hyperplasia is characterized by densely packed lobules and hyperplastic acini lined by epithelial cells which generally retain their normal cuboidal appearance (Figure 2Bi, ii, iii, Figure S2A in Additional file 1). Some acini may be filled with epithelial cells, but are not notably expanded in size (Figure 2B, black arrows). Mammary gland adenomas are characterized by marked epithelial proliferation that fills and markedly expands the acini and ducts additionally; the cells have slight cellular atypia (Figure 2B iv, v, vi, Figure S2B in Additional file 1). Mammary carcinomas are characterized by solid sheets of cells with little or no acinar architecture remaining (Figure 2B vii, viii, ix, Figure S2C in Additional file 1). The neoplastic cells have cellular and nuclear atypia, numerous mitotic figures, and frequently demonstrate invasion into the surrounding stroma (Figure 2B vii, viii, ix, Figure S2C in Additional file 1). Histologic evaluations of tumors revealed these features and were identified in all PyMT mice regardless of Mena genotype (Figure 2B).

Staging of tumors from PyMT Mena WT, Het and Null mice at 10 weeks of age showed that by 10 weeks 80% of Mena WT and Het mice had at least one tumor or parts of a tumor that had progressed to carcinoma (Figure 2C). Neither Mena WT nor Het mice had tumors that could be classified as hyperplastic due to the presence of more advanced stages of progression within these tumors (Figure 2C). Twenty percent of both genotypes of mice had tumors staged as adenoma (Figure 2C). Interestingly, 21% of Mena Null mice had mammary tumors that were staged as hyperplastic, 43% that were staged as adenoma and only 36% that were staged as carcinoma (Figure 2C). Additionally, we observed the presence of normal tissue in all animals (Mena WT, Het and Null) regardless of tumor stage. Thus, deficiency of Mena delays but does not prevent progression to malignancy (Figure 2C).

### Deficiency of Mena dampens EGF-induced in vivo invasion and motility

**Figure 3:**
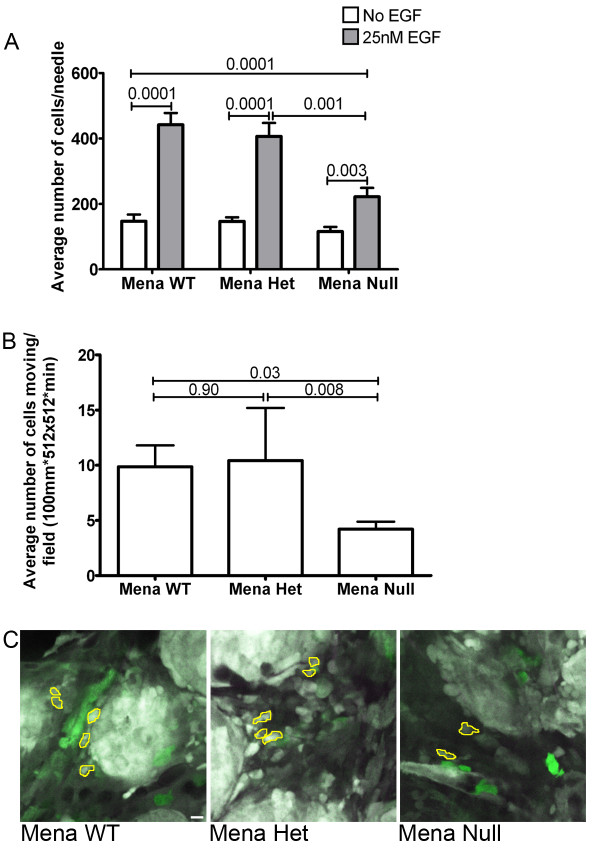
**Deficiency of Mena dampens EGF-induced *in vivo *invasion and motility**. **A**. *In vivo *invasion assay of PyMT mice with given Mena genotype. White bars indicate average number of tumor cells that invaded in the absence of EGF, gray bars indicate average number of tumor cells that invaded in the presence of 25 nM EGF. *P*-values listed above bars. N = 12 assays/genotype/condition. Error bars indicate SEM. **B**. Average number of cells moving/field quantified from intravital imaging of PyMT Mena WT, Het and Null mice. 30 to 50 fields analyzed from 10 mice per genotype. Error bars = SEM. **C**. Multiphoton microscopy of tumor cells moving within the primary tumors of PyMT Mena WT, Het and Null mice. Images taken at 60× over the course of 30 minutes. Yellow outlines indicate cells that are moving (refer to Additional files 2, 3, 4). White = CFP tumor cells, Green = GFP macrophages, Black = tumor stroma and vasculature. Scale bar = 50 μm.

An early step in metastatic progression is invasion out of the primary tumor and into the surrounding stroma [1]. Therefore, we used an *in vivo *invasion assay to score EGF induced invasion [49]. We found that Mena Null mice had significantly decreased *in vivo *invasion as compared to Mena WT and Het mice (Figure 3A). The requirement of Mena for invasion observed above is consistent with the overexpression of Mena isoforms which leads to increased invasion (Figure S3 in Additional file 1) and [23].

Another measure of invasive potential is cell migration within and out of the primary tumor into the stroma. Prior work showed increased motility in orthotopic mammary tumors derived from injection of MTLn3 rat adenocarcinoma cells forced to express Mena [23]. We used intravital multiphoton microscopy to investigate the effect of Mena deficiency on tumor cell motility *in vivo *in PyMT Mena WT, Het and Null mice. The average number of tumor cells moving per field was significantly decreased in Mena Null mice as compared to both Mena WT and Het mice (Figure 3B, C Additional files 2, 3, 4).

### Deficiency of Mena decreased intravasation and metastasis to the lung

**Figure 4:**
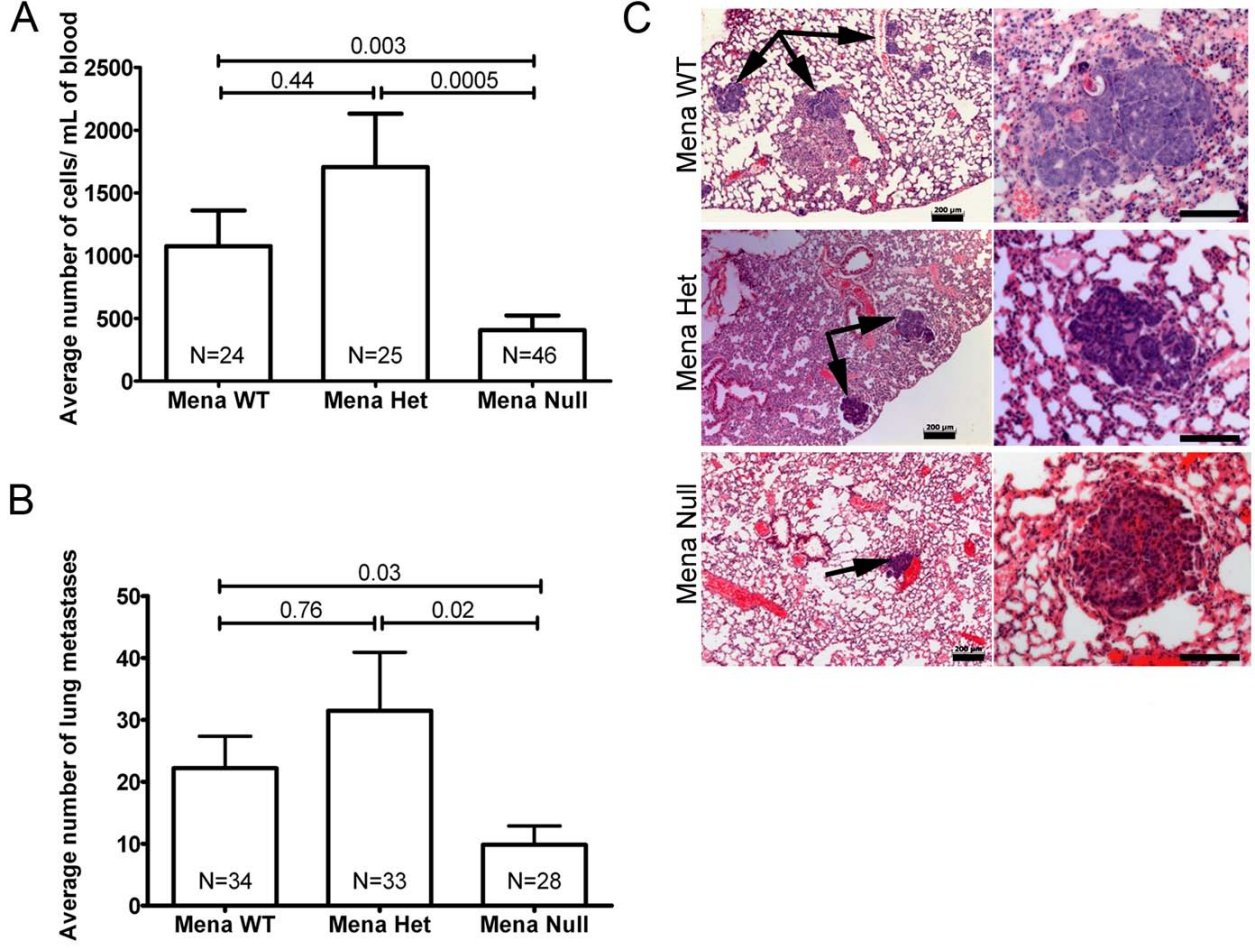
**Deficiency of Mena decreases intravasation and metastasis to the lungs**. **A**. Average number of circulating tumor cells in the blood of PyMT animals staged with carcinoma. Number of mice used of each genotype listed within bars. *P*-values listed above bars. Error bars indicate SEM. **B**. Average number of spontaneous lung metastases in PyMT animals staged with carcinoma. Number of mice used of each genotype listed within bars. *P-*values listed above bars. Error bars indicate SEM. **C**. Immunohistochemistry of lung metastases (indicated with black arrows) from the lungs of Mena WT, Het and Null mice. Images in the left column were taken at 50× (Scale bar = 200 μm), images in the right hand column were taken at 100× (Scale bar = 100 μm).

The next step of metastasis, intravasation into blood vessels, was measured by evaluation of the number of circulating tumor cells in a clonogenic assay since in PyMT mammary tumors, the number of circulating tumor cells has been correlated with intravasation [2]. In fact, Mena Null mice had significantly fewer circulating tumor cells as compared to Mena WT and Het mice, which were not significantly different from each other (Figure 4A).

Previous studies show that the forced expression of Mena in orthotopic mammary tumors derived from tumor cell injection into the mammary gland of SCID mice, significantly increased metastasis to the lung [23]. Thus, we hypothesized that deficiency of Mena would reduce the number of metastases to the lung. Evaluation of the number of lung metastases in PyMT from Mena WT, Het and Null mice, whose tumors were stage matched for carcinoma, showed that Mena Null mice had significantly fewer lung metastases as compared to Mena WT and Het mice (Figure 4B). Additionally, there were no differences observed in appearance (size and location) of lung metastases (Figure 4C).

These findings suggest that deficiency of Mena significantly decreases metastasis by affecting invasion, intravasation and motility within the primary tumor.

### Mena Null xenografts show decreased intravasation and lung metastasis

**Figure 5:**
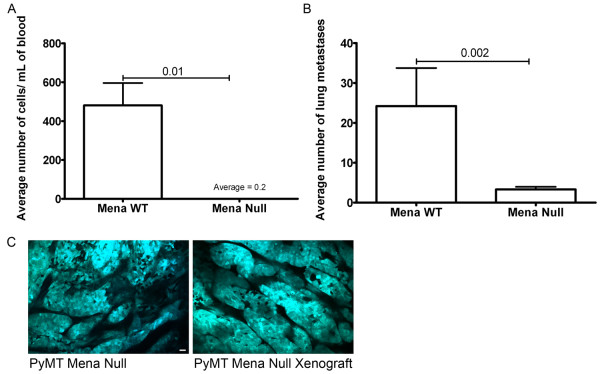
**Tumors from orthotopic transplantation of Mena Null cells show decreased intravasation and lung metastasis**. **A**. Average number of circulating tumor cells in the blood of SCID mice injected with PyMT Mena WT, and Null tumor cells. N = 10 mice/genotype. *P-*values listed above bar. Error bars indicate SEM. **B**. Average number of spontaneous lung metastases in SCID mice injected with PyMT Mena WT, and Null tumor cells. N = 10 mice/genotype. *P*-values listed above bar. Error bars indicate SEM. **C**. Multiphoton microscopy of primary tumors of SCID mice injected with PyMT Mena WT, and Null tumor cells. Images taken at 20×. Blue = CFP tumor cells, Black = tumor stroma and vasculature. Scale bar = 50 μm.

Mena is expressed in both stromal and tumor cells, raising the formal possibility that loss of Mena reduced metastasis through an effect on the tumor microenvironment rather than the tumor cells. To determine if the altered metastasis in Mena deficient animals resulted from defects intrinsic to the tumor cells, we isolated tumor cells from Mena Null or WT animals and transplanted them into SCID mice. The mammary glands of SCID mice were injected with Mena WT or Null tumor cells isolated by FAC sorting from the respective PyMT mammary tumors. Mice growing orthotopic tumors derived from injection of Mena Null cells showed significantly decreased numbers of circulating tumor cells (Figure 5A) and lung metastases (Figure 5B). For additional validation of this methodology we used intravital multiphoton microscopy to compare the primary tumor morphology of tumors derived from injection of PyMT tumor cells versus that of spontaneously growing tumors from PyMT animals. Surprisingly, we found that the xenograft tumors had a similar morphology to the spontaneously growing tumors from PyMT animals (Figure 5C). This observation suggests that this method provides a reliable recapitulation of the PyMT tumor. Overall, these data support the conclusions that deficiency of Mena in tumor cells is responsible for the significant decrease in intravasation and lung metastasis observed in PyMT Mena Null mice.

Given that a deficiency of Mena had such a profound effect on intravasation as compared to Mena WT mice (Figures 4A and 5A) we investigated if there were any differences in vascular density. Immunohistochemical staining of blood vessels was performed on primary tumors from PyMT Mena WT and Null mice and we found that Mena Null mice had significantly fewer vessels located throughout the tumors as compared to tumors from Mena WT mice (Figure S4A in Additional file 1). Immunohistochemical staining of blood vessels was also performed on primary tumors from xenograft mice derived from injection of tumor cells isolated from PyMT Mena WT and Null mice and we found that there was no difference in vascular density in Mena Null tumors as compared to Mena WT (Figure S4B in Additional file 1). Lastly, immunohistochemical staining of blood vessels performed on primary tumors from xenograft mice derived from injection of MTLn3 and MTLn3-Mena overexpressing tumor cells revealed no difference in vascular density in Mena overexpressing tumors as compared to MTLn3 control (Figure S4C in Additional file 1). Hence, the large difference in intravasation score observed in these different cases is not explained by differences in microvessel density alone.

### Mena deficiency reduces terminal end bud formation and branching during mammary gland development

**Figure 6:**
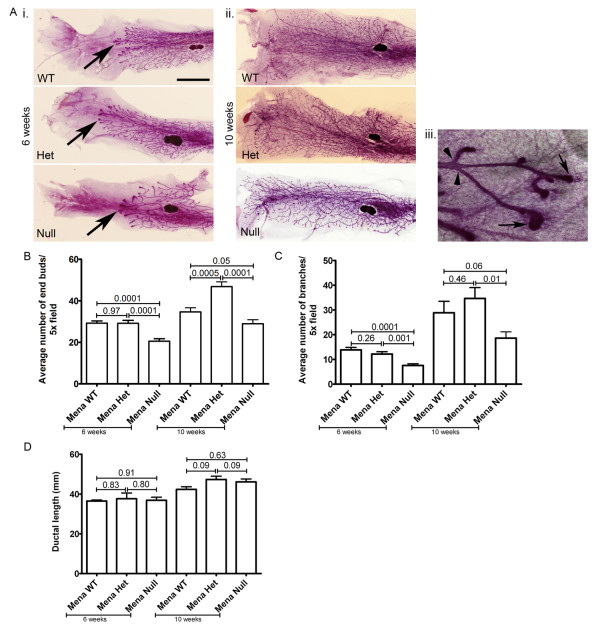
**Deficiency of Mena alters mammary gland development**. **A**. Representative images of mammary gland whole mounts from PyMT Mena WT, Het and Mena Null mice at 6 weeks (i) and 10 weeks (ii) of age. Scale bar = 1 cm. Enlarged area of six-week whole mount mammary glands shown in iii. In iii = whole mount mammary gland from PyMT Mena WT mouse. Black arrows point to examples of terminal end buds. Black arrowheads point to examples of branches. Images taken at 10×. **B**. Quantification of terminal end buds from mammary gland whole mounts. Examples of terminal end buds counted are shown in Figure 6A by the black arrows. N = 5 animals per genotype at each time point. *P*-values listed above bars. Error bars = SEM. **C**. Quantification of mammary duct branching quantified under 5×. Examples of branches counted are shown in Figure 6A iii by the black arrowheads. N = 5 fields/animal, 10 animals per genotype. *P*-values listed above bars. Error bars = SEM. **D**. Quantification of the length of the three longest ducts from mammary gland whole mounts. N = 10 animals per genotype. *P*-values listed above bars. Error bars = SEM.

Post-natal mammary gland development is commenced by formation and outgrowth of multilaminate epithelial structures called terminal end buds (TEB) (black arrows, Figure 6Aiii) [34]. These bifurcate to give a primary branched structure that then attains secondary branches along the primary ductal tree (black arrow heads, Figure 6Aiii). During this process of branching morphogenesis, the TEBs invade the surrounding fatty stroma in response to signals from cells in the surrounding microenvironment [34]. Given that Mena is expressed in normal mammary ductal epithelial cells (Figure S5 in Additional file 1), and that Mena deficiency leads to decreased *in vivo *invasion in PyMT tumor bearing mice (Figure 3A), we sought to determine if the deficiency of Mena affects invasive stages of normal mammary gland development. Thus, we investigated TEB formation, branching and ductal growth in mammary glands of otherwise normal mice that do not express the PyMT oncogene. Examination of mammary gland whole mounts from Mena WT, Het and Null mice at 6 weeks (Figure 6Ai) and 10 weeks (Figure 6Aii) of age showed a significant decrease in the number of TEBs in Mena Null mice as compared to Mena WT and Het (Figure 6Ai, ii and 6B). Additionally, investigation of these same whole mounts showed significantly decreased ductal branching in Mena Null as compared to Mena WT and Het mice at six weeks (Figure 6C). However, at 10 weeks ductal branching in Mena Null mice was not significantly different from Mena WT (Figure 6C). Given that Mena expression does not affect tumor growth [23], we hypothesized that duct length would be unaffected and indeed we found it was unaffected by Mena deficiency at both 6 and 10 weeks of age (Figure 6D). Interestingly, a deficiency of Mena delays but does not prevent development of TEBs and ductal branching (Figure 6B, C) similar to the delay seen during tumor progression in PyMT Mena Null mice (Figure 2). These data suggest that Mena's role in developmental processes may be similar to its role during tumor progression. Another interesting finding is that Mena Het mice have significantly increased TEB formation at 10 weeks as compared to Mena WT (Figure 6B). Given that different aspects of breast development have been shown to play a role in breast disease [50-52], it is possible that the changes observed in TEB formation in Mena Null and Mena Het mice could be correlated with tumor latency; a decrease in TEB formation in Mena Null mice could lead to increased tumor latency (Figure 1A) slowing progression (Figure 2C), while an increase in TEB formation in Mena Het mice could lead to decreased latency (Figure 1A) and thus enhanced progression.

**Figure 7:**
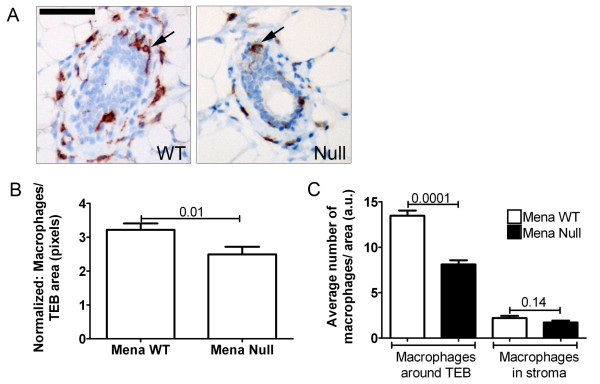
**Deficiency of Mena decreases macrophage recruitment to terminal end buds**. **A**. Immunohistochemistry of terminal end buds of mammary glands from PyMT Mena WT, and Null mice. Blue = h ematoxilin and brown = F4/80+ macrophages (examples identified by black arrows). Scale bar = 50 μm. **B**. Quantification of macrophage number per surface unit of terminal end bud. N = 10 animals per genotype. *P*-values listed above bars. Error bars = SEM. **C**. Quantification of the number of macrophages around terminal end buds and the number of macrophages in surrounding stroma in 200× field in both Mena WT (white bars) and Mena Null (black bars) animals. N = 5 animals per genotype. *P*-values listed above bars. Error bars = SEM.

Interactions between epithelial and stromal cells contribute to the development of the epithelial ductal tree during mammary gland development [34,53,54], specifically, macrophages play a role in tissue remodeling during development and in normal tissue homeostasis [40,55]. Additionally, studies have shown that elimination of macrophages in PyMT mice significantly decreases lung metastasis [56] and EGF induced *in vivo *invasion [3]. Previously, we have shown that expression of Mena and Mena^INV ^in tumor cells sensitizes cells to stimulation by EGF during macrophage mediated *in vivo *invasion [23] and transendothelial migration [57]. We have also shown that increased *in vivo *invasion and streaming tumor cell motility in Mena^INV ^expressing tumor cells is dependent on macrophages [57]. We hypothesize that expression of Mena enhances tumor cell/macrophage paracrine interactions and that elimination of Mena will significantly decrease macrophage dependent processes. Previous studies suggest that macrophages are required for the formation of TEBs and branching of associated ducts [40]. Therefore, given that Mena Null mice show a defect in terminal end bud formation and branching, we measured the number of macrophages surrounding the TEBs at six weeks of age. The number of macrophages was evaluated for a standardized TEB area to account for differences in TEB size (Figure 7B). Macrophage density was also counted in a given area surrounding TEBs and in the stroma (not surrounding TEB) to determine if macrophage recruitment to TEB was specifically affected (Figure 7C). We found that there are fewer macrophages per TEB area in Mena Null mice vs. Mena WT (Figure 7A, B). Additionally, the number of macrophages recruited to TEB is significantly reduced as compared the number of stromal macrophages in Mena Null vs. Mena WT mice (Figure 7C). These data confirm that a deficiency of Mena specifically leads to decreased recruitment of macrophages around the TEBs. These results suggest that Mena may be necessary for mammary gland differentiation and could play a direct or indirect role in macrophage mediated invasion *in vivo*.

## Discussion

Through the use of newly developed technologies including, high density mircroarray based expression profiling, intravital imaging and the collection of live invasive tumor cells from primary tumors, transiently expressed genes, such as Mena were identified as modulators of metastasis [8]. Such findings support the theory of metastasis whereby a population of tumor cells exhibits metastatic capabilities very early in tumor progression as a result of transient changes in gene expression. The Invasion Signature is a set of transiently regulated genes in metastatic tumor cells that control their chemotactic and migratory behavior; Mena has been identified as a master regulator of cell motility pathways in the Invasion Signature [8,23]. The upregulation of Mena in rat, mouse, and human mammary tumors [5,11,12], use of Mena in the prognostic marker TMEM [12], and Mena's significant role in the regulation of metastatic progression [19,21,23], render Mena an interesting therapeutic target. Our findings indicate that elimination of Mena activity could be a useful goal for therapy of metastatic disease.

### Mena deficiency delays tumor progression and decreases invasion, intravasation, and metastases

Our results demonstrate that loss of Mena in a transgenic mouse model of breast carcinoma decreases metastatic potential due to decreased invasion and intravasation. Importantly, Mena Null mice remain healthy for longer despite their equally large tumor burden as compared to Mena WT and Het mice. While a delay in onset of tumor development is observed, this does not seem to be a major contributing factor affecting the drastic increase in survival given that Mena Null mice still show histological progression to invasive carcinoma. Thus, the dramatic increase in survival of Mena Null mice is most likely associated with the significant decrease in formation of lung metastases. We propose that the increase in tumor latency may be a result of decreased ductal branching and TEB formation identified in Mena Null mammary gland development. Additionally, the decrease in tumor latency seen in Mena Het animals could be attributed to the increase in TEB identified during mammary gland development. This phenomenon will be investigated in future studies.

Given that metastasis is responsible for the majority of cancer related deaths [58], it is important to determine the mechanisms involved in this process. Previous studies in PyMT mice illustrate the contribution of the microenvironment to metastatic potential and describe a paracrine loop whereby invasion, intravasation and metastasis involve signaling between macrophages and tumor cells where these cells secrete EGF and CSF1 respectively [3,4]. Our findings illustrate that a deficiency in Mena leads to blunted EGF induced *in vivo *invasion, intravasation and lung metastases.

Previous studies have shown that the motility of tumor cells correlates with their ability to intravasate and that both processes require the participation of macrophages [3]. Multiphoton intravital microscopy is a powerful tool used to study tumor cell behavior within primary tumors and enables the visualization of differences in motility within the tumor microenvironment [47]. We demonstrate that tumor cells in Mena Null mice move significantly less than tumor cells in Mena WT and Het mice, another contributing factor toward decreased metastatic potential. These findings are consistent with an increase in movement and velocity in MTLn3 rat adenocarcinoma cells forced to express Mena isoforms [23]. Additionally, given that there is no difference in vascular density in xenograft tumors derived from injection of Mena Null and Mena WT cells obtained from PyMT animals or in xenografts derived from MTLn3 Mena overexpressing cells, we conclude that the decrease in intravasation observed in both transgenic PyMT Mena Null mice and in Mena Null xenograft mice is the result of a Mena deficiency in tumor cells leading to a change in their intravasation phenotype and is not due to changes in vascular density. We hypothesize that the decrease in vasculature observed in transgenic PyMT Mena Null mice is related to the deficiency of Mena in endothelial cells and requires further investigation.

### Mena deficiency delays ductal branching and terminal end bud formation during mammary gland development

Control of tumor cell invasion has been linked to the reawakening and seizure of developmental programs by malignant cells [33,35]. Our findings, that loss of Mena decreases ductal branching and TEB formation (both considered to be invasive processes in the developing mammary gland), and blunts invasion during metastatic progression, suggests that Mena's role in developmental invasion is recapitulated during metastatic progression. Interestingly, during nervous system development, Ena/VASP proteins are required for normal response to the axon guidance factors SLIT and Netrin [30,59]. Both SLIT and Netrin have been shown to play an important role in mammary gland morphogenesis [32,60,61] and in regulation of cell invasion across basement membranes [62]. Thus, we speculate that Mena's affects on invasion during development and metastasis may involve SLIT and Netrin. Future studies will evaluate this hypothesis and determine the mechanism behind the observations reported here. Additionally in humans, intraductal spreading can be observed in malignant lesions classified as ductal carcinoma *in situ *(DCIS) and may be related to the process of ductal branching during development [63,64]. Since DCIS shows no evidence of invasion into surrounding stroma, a decrease in ductal branching in Mena Null mice could contribute to the delay in histological tumor progression but not metastatic progression. Additionally, intraductal spreading as identified in human cancer was not observed in the PyMT model of invasive breast carcinoma (Rani Sellers, personal communication) therefore, it will be interesting to determine if Mena deficiency affects this phenomenon in a different model.

Our finding that fewer macrophages are recruited to TEBs in the developing mammary gland of Mena Null mice, and our previous findings showing macrophage dependent *in vivo *invasion, intravasation and motility of Mena expressing cells [57], suggests that Mena is involved in a signaling loop with macrophages during development as well as during metastatic progression. Additionally, macrophages lack detectable Mena expression, but they do express the related Ena/VASP family members VASP and EVL (Unpublished observations, FBG). Thus tumor cell/macrophage interactions, or lack thereof, as described above, may be due to a Mena deficiency in tumor cells specifically (as supported by xenotransplant studies described in Figure 5). Further studies will be conducted to investigate this hypothesis.

## Conclusions

Deficiency of Mena in the PyMT transgenic model of breast carcinoma increases tumor latency and decreases the rate of tumor progression to the histologic stage of carcinoma. Significant reductions in EGF induced *in vivo *invasion, intravasation and motility are strongly correlated with the decrease in lung metastasis and morbidity seen in Mena Null mice. This striking decrease in metastasis is recapitulated by the implantation of Mena Null tumor cells into a mouse host that is wild type for Mena showing a tumor cell autonomous effect. Additionally, Mena deficiency in non-tumor bearing mice leads to defects in invasive stages of mammary gland development such as terminal end bud formation and ductal branching, raising the intriguing possibility that Mena is part of a developmental program that is re-activated during metastatic progression. Given that loss of Mena is compatible with viability, we propose that inhibition of Mena could be used as a treatment for metastatic disease in breast cancer patients.

## Abbreviations

CSF1: colony stimulating factor 1; EGF, epidermal growth factor; EGFP, enhanced green fluorescent protein; FACs, fluorescence activated cell sorting; Het, heterozygote; IHC, imm, nohistochemistry; IF, immunofluorescence; PyMT, polyoma Middle T antigen; SCID, severe combined immunodeficiency; TEB, terminal end bud; TMEM, tumor microenvironment for metastasis; WT, wild type.

## Competing interests

The authors declare that they have no competing interests.

## Authors' contributions

ETR carried out the majority of the animal studies including: evaluation of tumor latency and morbidity, tumor staging, blood burden, intravital imaging, and evaluation of metastasis. YW was responsible for breeding and genotyping of all animals, assistance in evaluation of staging, metastasis and motility. JBW and JC were responsible for overseeing all intravital imaging and analysis and made intellectual contributions to experimental design. WW, JW, JC and JWP were responsible for the generation of PyMT mice. FBG and JC were responsible for conception of the project and oversight of all experiments and training of certain participants.

## Supplementary Material

Additional file 1 **Figure S1: Average number of tumors per mouse at 16 weeks of age**. N = 25 mice/genotype. Error bars indicate SEM. *P*-values by student's *t*-test are listed above columns. Figure S2: Imunohistochemistry images (400×) of mammary tumors from Mena WT mice at 10 weeks of age. **A**. Hyperplasia: densely packed lobules and hyperplastic acini lined by epithelial cells which generally retain their normal cuboidal appearance. Some acini may be filled with epithelial cells, but are not notably expanded in size. **B**. Adenoma: marked epithelial proliferation which fill and expand the acini and ducts. Cells have slight cellular atypia (box). **C**. Carcinomas: solid sheets of cells with little or no acinar architecture remaining. Neoplastic cells have cellular and nuclear atypia (box), numerous mitotic figures (arrow), and frequently demonstrate invasion into the surrounding stroma. Figure S3: *In vivo *invasion assay of tumor cells from tumors in xenograft mice derived from injection of MTLn3 or MTLn3-Mena overexpressing cells. Bars indicate the average ratio of: tumor cells collected in 25 nM EGF containing needles/tumor cells collected in control needles (containing no EGF). *P*-value listed above bars. N = 12 assays/genotype/condition. Error bars indicate SEM. Figure S4: **A**. Average number of blood vessels in primary tumors from PyMT Mena WT and Null mice. Error bars indicate SEM. P values listed above bars. **B**. Average number of blood vessels in xenograft primary tumors derived from injection of PyMT Mena WT and tumor cells. Error bars indicate SEM. P values listed above bars. **C**. Average number of blood vessels in xenograft primary tumors derived from injection of MTLn3 control and MTLn3-Mena over-expressing tumor cells. Error bars indicate SEM. P values listed above bars. Figure S5: IHC of normal/non-tumor bearing mammary glands from Mena WT mice at 10 weeks of age stained with antibody against Mena (brown) and counterstained with Hematoxylin (purple). Image taken at 40×. Scale bar = 20 μm. Click here for file

Additional file 2 **Tumor cell motility in PyMT Mena WT mice**. Multiphoton microscopy of tumor cells moving within the primary tumors of PyMT Mena WT mice. Images taken at 60× over the course of 30 minutes. White = CFP tumor cells, Green = GFP macrophages, Black = tumor stroma and vasculature. Click here for file

Additional file 3 **Tumor cell motility in PyMT Mena Het mice**. Multiphoton microscopy of tumor cells moving within the primary tumors of PyMT Mena Het mice. Images taken at 60× over the course of 30 minutes. White = CFP tumor cells, Green = GFP macrophages, Black = tumor stroma and vasculature. Click here for file

Additional file 4 **Tumor cell motility in PyMT Mena Null mice**. Multiphoton microscopy of tumor cells moving within the primary tumors of PyMT Mena Null mice. Images taken at 60× over the course of 30 minutes. White = CFP tumor cells, Green = GFP macrophages, Black = tumor stroma and vasculature. Click here for file
