# Supplementary figures and images for: Mena deficiency delays tumor progression and decreases metastasis in polyoma middle-T transgenic mouse mammary tumors
Source: Breast Cancer Res. 2010 Nov 25;12(6):R101. doi: 10.1186/bcr2784 (PMC3046446; doi:10.1186/bcr2784)

**Supplemental Data**


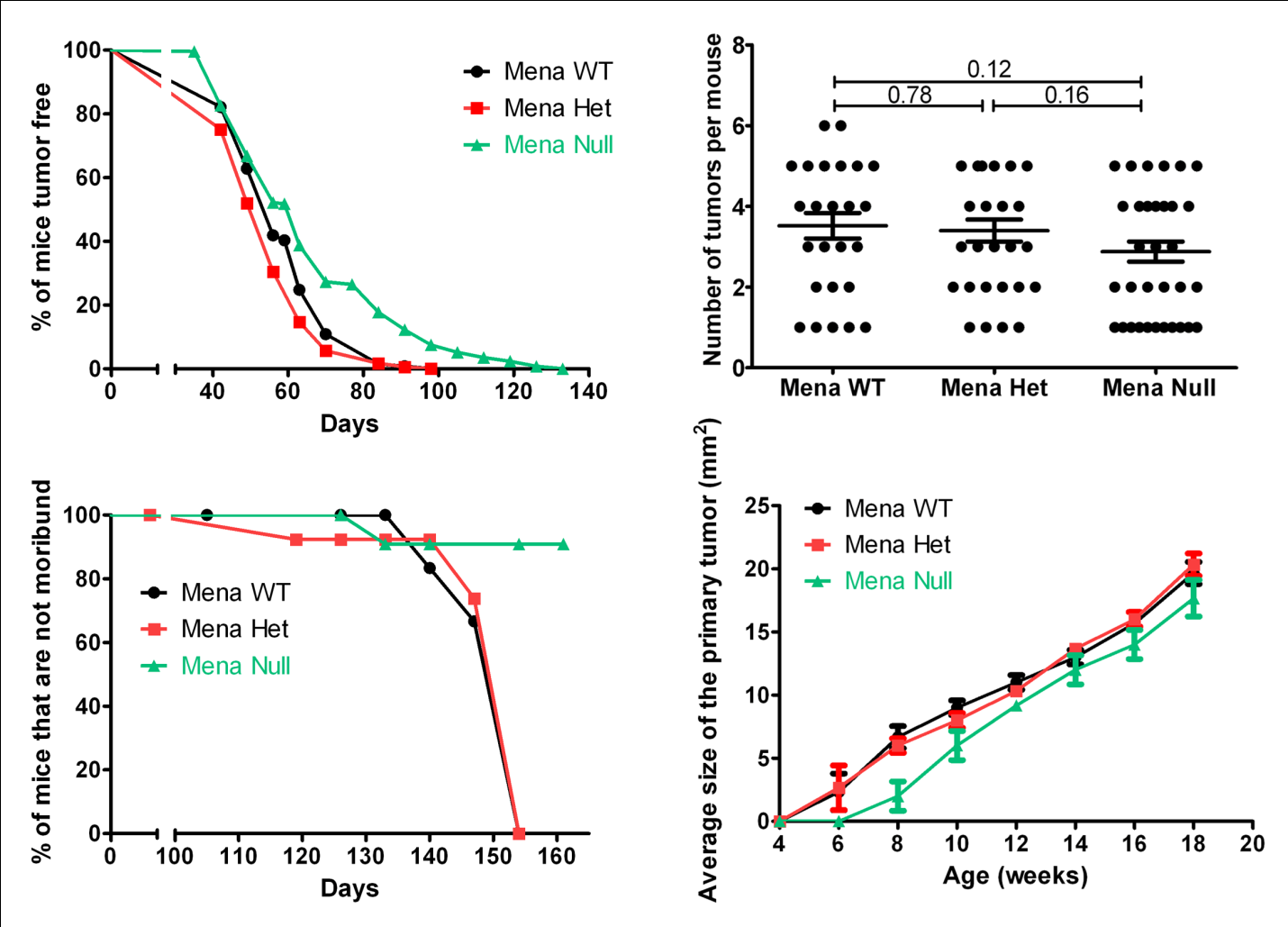


Figure S1

**
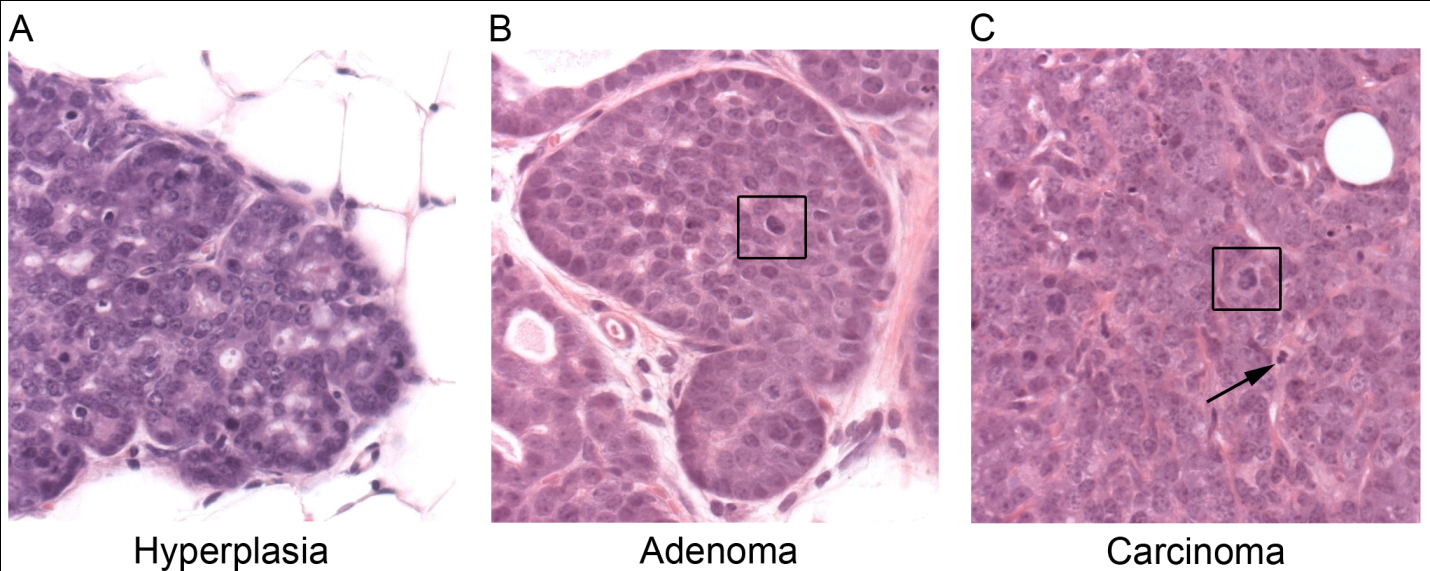
**

Figure S2


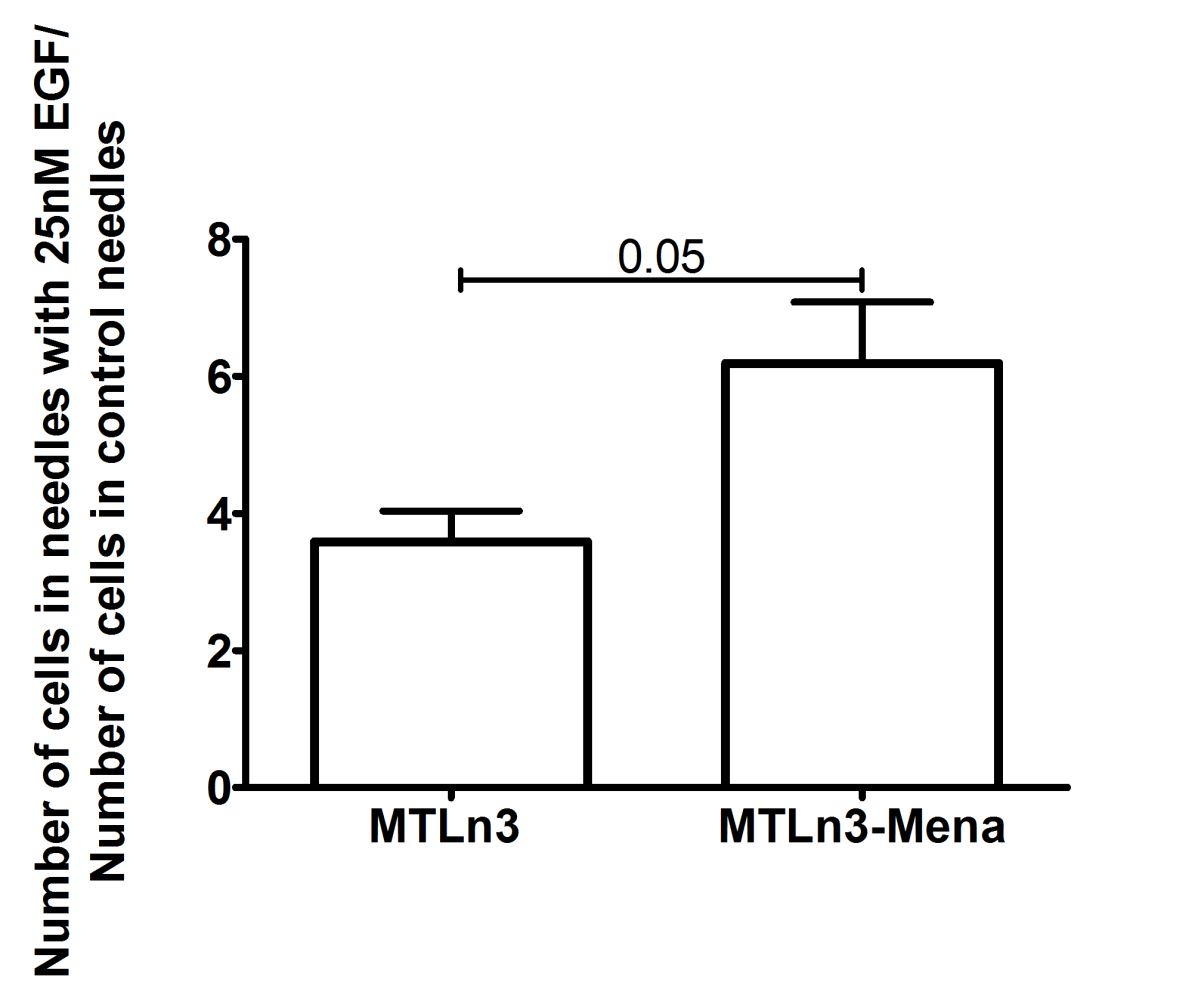


Figure S3


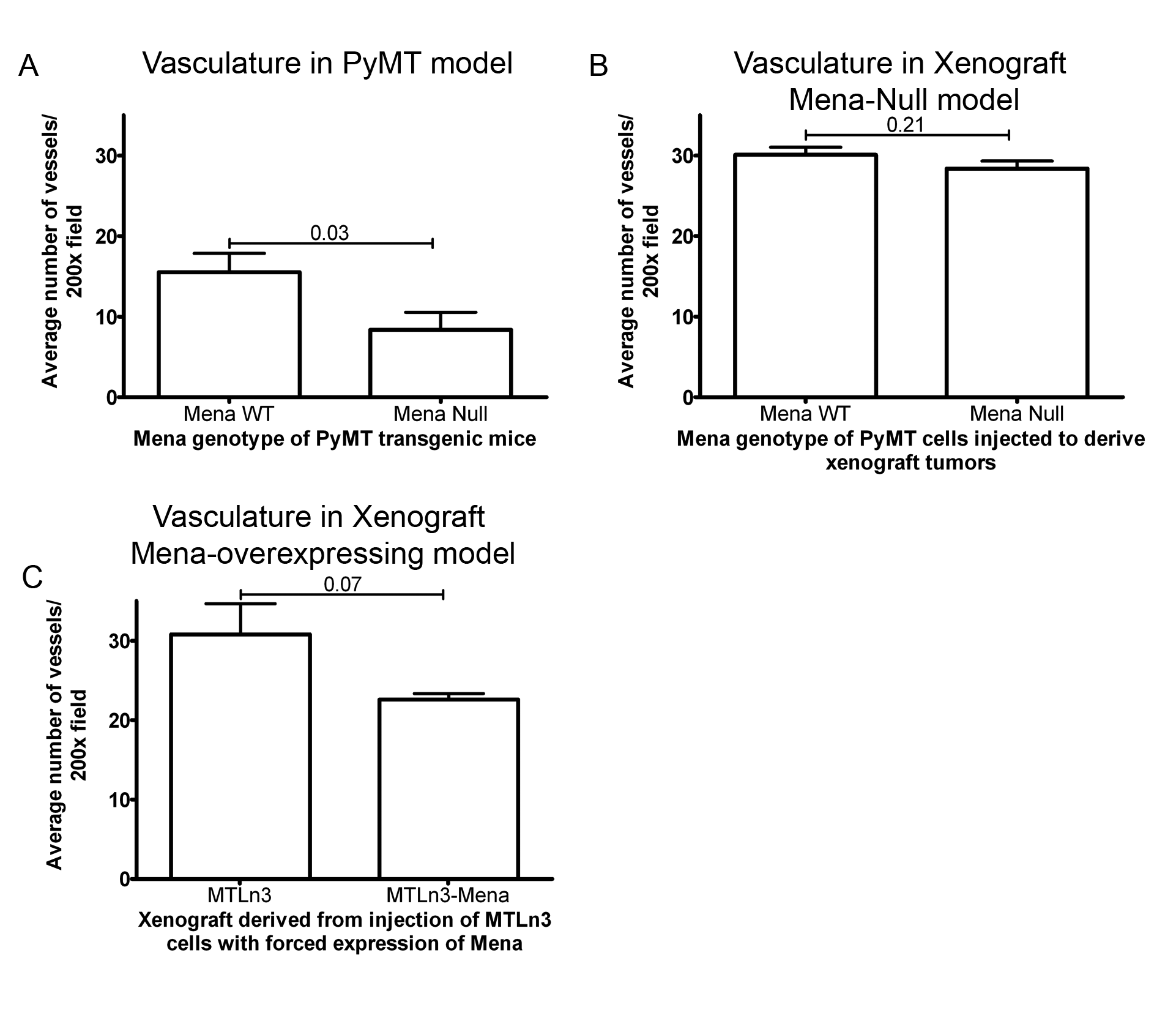


Figure S4


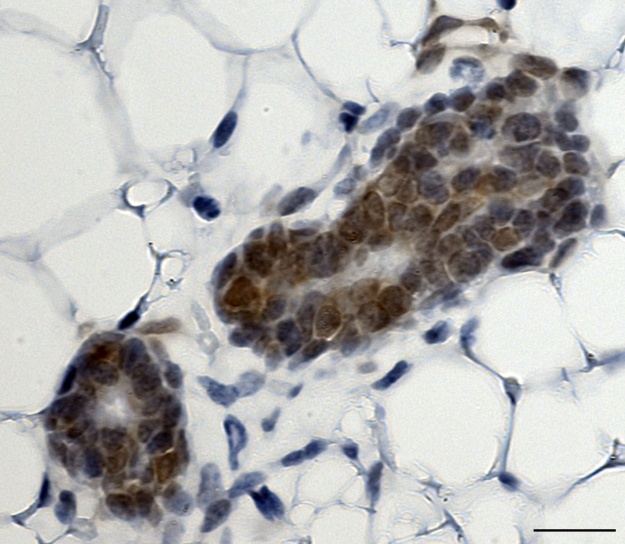


Figure S5

Supplement: Additional file 1 — Figure S1: Average number of tumors per mouse at 16 weeks of age. N = 25 mice/genotype. Error bars indicate SEM. P-values by student's t-test are listed above columns. Figure S2: Imunohistochemistry images (400×) of mammary tumors from Mena WT mice at 10 weeks of age. A. Hyperplasia: densely packed lobules and hyperplastic acini lined by epithelial cells which generally retain their normal cuboidal appearance. Some acini may be filled with epithelial cells, but are not notably expanded in size. B. Adenoma: marked epithelial proliferation which fill and expand the acini and ducts. Cells have slight cellular atypia (box). C. Carcinomas: solid sheets of cells with little or no acinar architecture remaining. Neoplastic cells have cellular and nuclear atypia (box), numerous mitotic figures (arrow), and frequently demonstrate invasion into the surrounding stroma. Figure S3: In vivo invasion assay of tumor cells from tumors in xenograft mice derived from injection of MTLn3 or MTLn3-Mena overexpressing cells. Bars indicate the average ratio of: tumor cells collected in 25 nM EGF containing needles/tumor cells collected in control needles (containing no EGF). P-value listed above bars. N = 12 assays/genotype/condition. Error bars indicate SEM. Figure S4: A. Average number of blood vessels in primary tumors from PyMT Mena WT and Null mice. Error bars indicate SEM. P values listed above bars. B. Average number of blood vessels in xenograft primary tumors derived from injection of PyMT Mena WT and tumor cells. Error bars indicate SEM. P values listed above bars. C. Average number of blood vessels in xenograft primary tumors derived from injection of MTLn3 control and MTLn3-Mena over-expressing tumor cells. Error bars indicate SEM. P values listed above bars. Figure S5: IHC of normal/non-tumor bearing mammary glands from Mena WT mice at 10 weeks of age stained with antibody against Mena (brown) and counterstained with Hematoxylin (purple). Image taken at 40×. Scale bar = 20 μm. [file bcr2784-S1.DOC]
